# Validation of a patient-specific system for mandible-first bimaxillary surgery: ramus and implant positioning precision assessment and guide design comparison

**DOI:** 10.1038/s41598-020-70107-w

**Published:** 2020-08-07

**Authors:** Giovanni Badiali, Mirko Bevini, Federica Ruggiero, Laura Cercenelli, Elisa Lovero, Elisabetta De Simone, Paola Rucci, Alberto Bianchi, Claudio Marchetti

**Affiliations:** 1 Azienda Ospedaliero-Universitaria di Bologna, Via Massarenti 9, Bologna, Italy; 2 Department of Biomedical and Neuromotor Sciences, University of Bologna, Bologna, Italy; 3 Department of General Surgery and Medical-Surgical Specialties, University of Catania, Catania, Italy

**Keywords:** Maxillofacial surgery, Oral surgery, Malocclusion, Three-dimensional imaging, Bone imaging, Fracture repair, Biomedical engineering

## Abstract

In orthognathic surgery, the use of patient-specific osteosynthesis devices is a novel approach used to transfer the virtual surgical plan to the patient. The aim of this study is to analyse the quality of mandibular anatomy reproduction using a mandible-first mandibular-PSI guided procedure on 22 patients. Three different positioning guide designs were compared in terms of osteosynthesis plate positioning and mandibular anatomical outcome. PSIs and positioning guides were designed according to virtual surgical plan and 3D printed using biocompatible materials. A CBCT scan was performed 1 month after surgery and postoperative mandibular models were segmented for comparison against the surgical plan. A precision comparison was carried out among the three groups. Correlations between obtained rami and plates discrepancies and between planned rami displacements and obtained rami discrepancies were calculated. Intraoperatively, all PSIs were successfully applied. The procedure was found to be accurate in planned mandibular anatomy reproduction. Different guide designs did not differ in mandibular outcome precision. Plate positional discrepancies influenced the corresponding ramus position, mainly in roll angle and vertical translation. Ramus planned displacement was found to be a further potential source of inaccuracy, possibly due to osteosynthesis surface interference.

## Introduction

Computer-assisted designed and manufactured (CAD/CAM) devices—widely known as Patient Specific Implants (PSIs)—have been increasingly adopted for orthognathic surgery, as per recent evidence in Literature of gaining better accuracy for virtual planning transfer to the patient (Gander 2015, Lin 2015)^1,2^. Several authors described and/or validated the use of PSIs for maxillary osteotomy and repositioning (Mazzoni 2015, Kraeima 2016, Suojanen 2016, Xue 2018)^3–6^. On the contrary, few authors—and more recently—described the use of PSIs for bilateral sagittal split osteotomy (BSSO) (Savoldelli, Li, Suojanen, Brunso)^7–10^, which appears consistent with the need for an accurate reproduction of the complex and partly uncontrolled movements of the condylar segment. Additionally, even fewer authors describe PSIs application in bimaxillary orthognathic surgery, but the conflict between potential inaccuracies of maxillary and mandibular CAD/CAM fixation has not been fully investigated (Li 2018, Suojanen 2017)^8,9^. The aim of this trial is to demonstrate that the mandible-first approach with BSSO-only CAD/CAM fixation could help overcome PSI inapplicability due to combined maxillary and mandibular fixation inaccuracies. The purpose of this paper is to measure the reproducibility of the digital planning in a cohort of patients treated with this approach. Specifically, we focused on the accuracy of the reproduction of the planned mandibular anatomy. Three different PSI positioning guide designs have been compared in terms of accuracy in virtual surgery plan transfer to the patient.

## Methods

We prospectively included 22 patients—seven males and fifteen females, mean age 26 (range 18–43)—undergoing orthognathic bimaxillary surgery at the Oral and Maxillofacial Surgery Unit of Sant’Orsola-Malpighi University Hospital (Bologna, Italy) between July 2017 and June 2019. Eight patients were diagnosed with skeletal class 2 deformity (one with combined facial asymmetry), ten were diagnosed with skeletal class 3 (six with combined asymmetry), three patients were diagnosed with class 1 facial asymmetry and one with anterior open bite. The present protocol was approved by the Sant’Orsola-Malpighi University Hospital ethics committee (approval number 238/2012/0/Disp PL02, amended 18/10/2016); the study conformed to the principles of the Declaration of Helsinki. Informed consent was obtained from all patients upon enrollment in the trial.

The study workflow consisted in the following steps: (1) data acquisition, (2) virtual planning, (3) computer-aided design and 3D printing of customized positioning guides and implants, (4) surgery and (5) Outcome evaluation.

### Data acquisition

Patients were enrolled when pre-operative orthodontic treatment was completed or when an adequate and stable final occlusion was achieved. All patients performed a pre-operative CBCT-scan (NewTom VGI Evo—*Cefla Group, Imola, Italy*), (24 × 19 cm FOV, 0.3 mm voxel) in a clinically-determined natural head position (NHP) and using a wax bite obtained in clinically-set condylar centric relation (with the aid of Dawson’s maneuver), 1 month prior to surgery.

Dental digital models were acquired using the CS 3600 intraoral scanner (*Carestream Health Inc, Rochester, NY, USA*) to produce stereolithographic output files (.STL) and obtain a virtual model of the dental arches. Digital models were 3D-printed using a stereolithographic printer (Form 2, *Formlabs Inc., Somerville, MA, USA*) and the final occlusion was manually determined. Models in final occlusion were re-scanned obtaining the corresponding digital model (.STL format).

### Virtual planning

**Figure 1:**
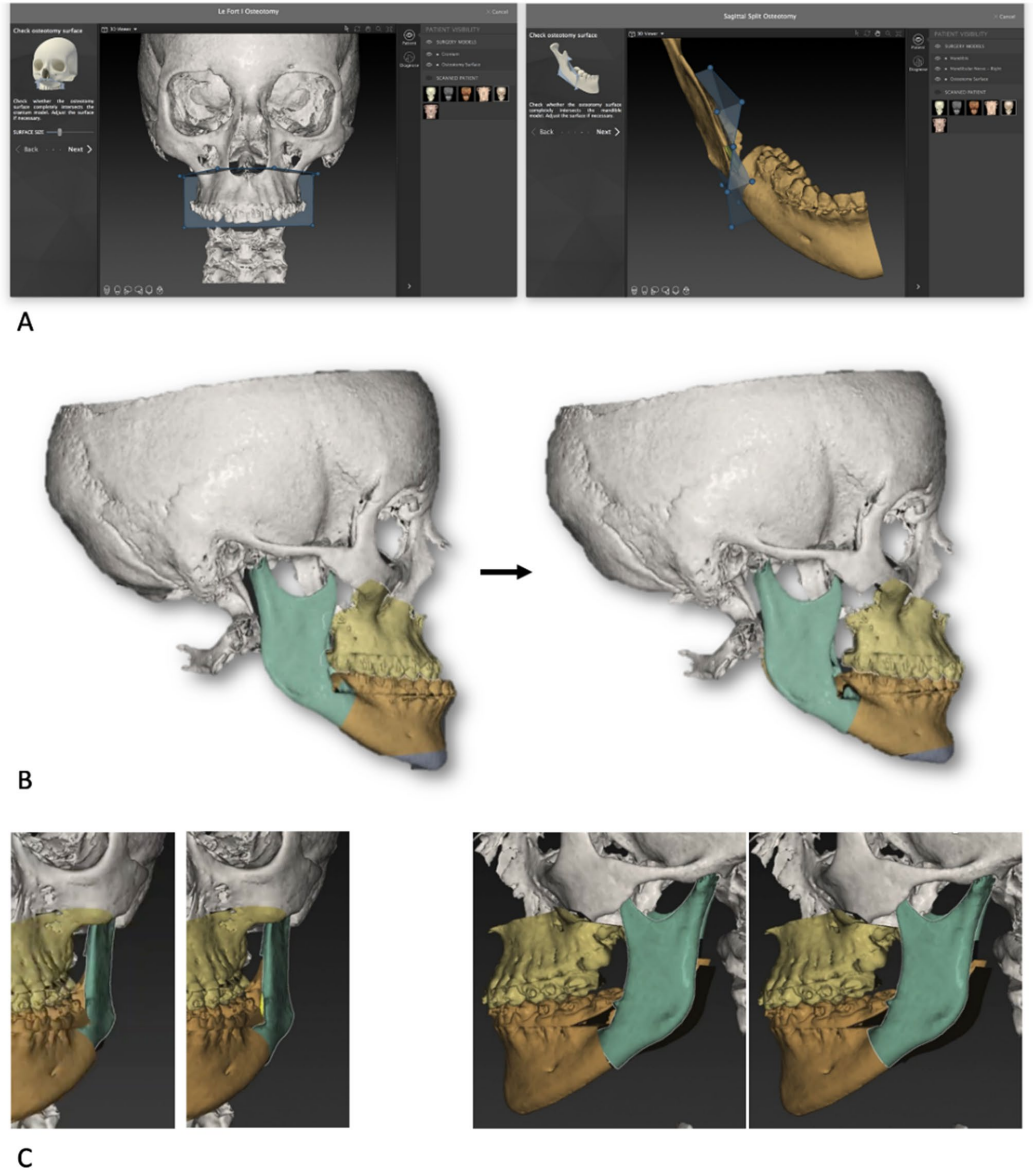
(**A**) Virtual osteotomies in IPS CaseDesigner. (**B**) Virtual surgery planning in IPS CaseDesigner. (**C**) Ramus position planning in IPS CaseDesigner. Single images joined in Adobe Photoshop 19 software (*Adobe Inc. San Jose, CA, U.S.*).

Cone-beam CT datasets (exported in DICOM format) and data from the intraoral scans were processed by the surgeon using IPS Case Designer software (*KLS Martin, Tuttlingen, Germany*). This software allowed to segment patient’s facial hard tissue and register dental digital models on the DICOM-segmented mesh with a semi-automatic algorithm. Moreover, it allowed to perform a three-dimensional cephalometric analysis, according to Swennen^11^, virtual BSSO was designed in order to replicate the expected achievable osteotomy (Fig. 1A). The surgical team realised the virtual surgical planning according to aesthetic parameters and cephalometric measurements (Fig. 1B). This allowed to plan the condyle/ramus fragment adjustments (Fig. 1C) pivoted on the geometrical center of the condylar head, with the following goals: (1) avoiding interference with the teeth-bearing fragment or (2) knowing unavoidable interference to be managed with selective additional osteotomies of the lingual fragment, (3) aligning the inferior borders on the sagittal plane; (4) obtaining the least possible condylar displacement compatible with the requested ramus adjustments. A couple of surgical splints (intermediate and final) was designed based on the virtual surgical plan. The mandibular procedure was designed as a potentially splint-less surgery, nevertheless surgical splints were produced in order to have a back-up solution in case of intraoperative failure of the system. Eventually, the intermediate splint was systematically used in order to keep the teeth-bearing fragment more stable during fixation.

### PSIs and positioning guides design and manufacture

**Figure 2:**
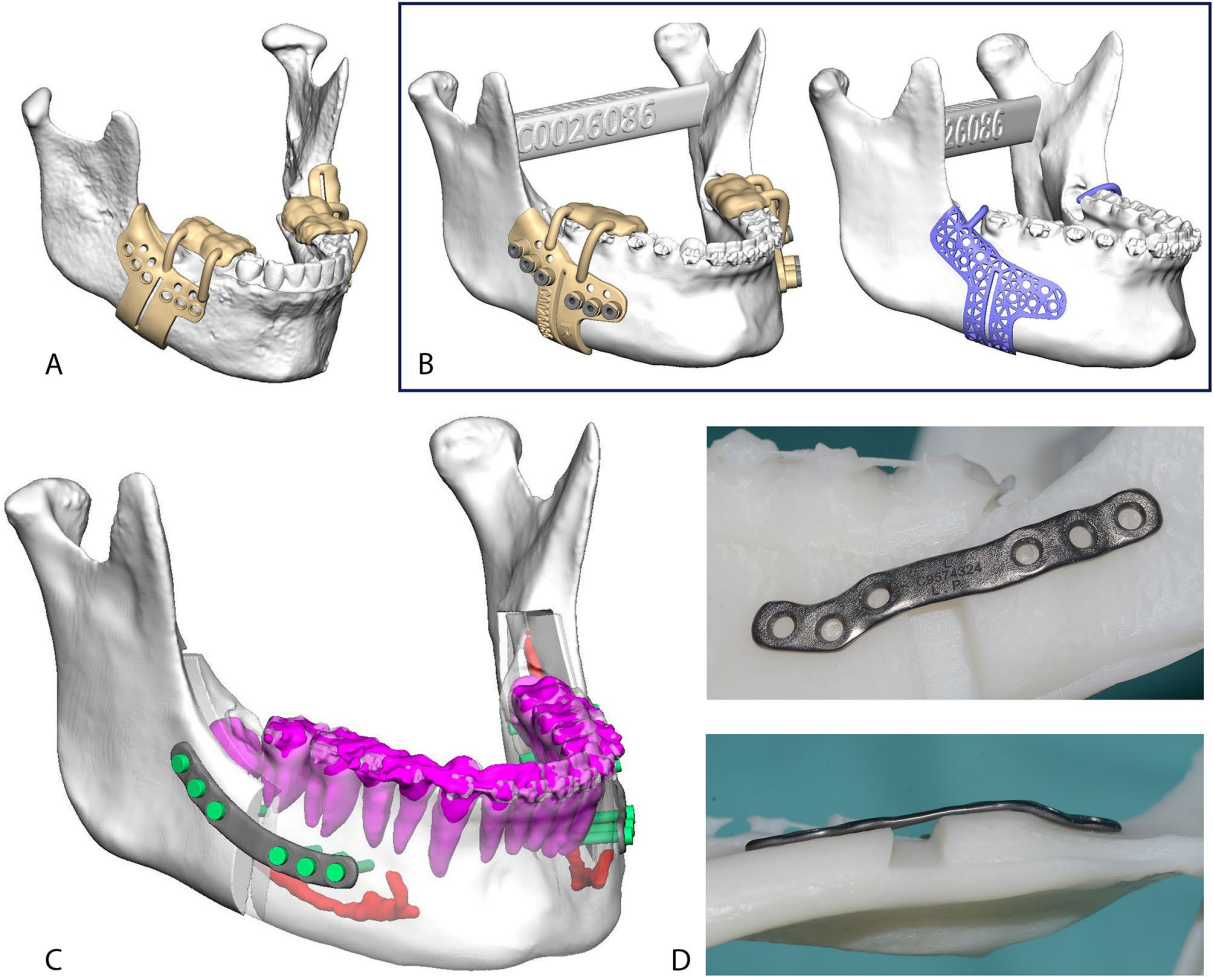
(**A**) Design A guide CAD rendering. (**B**) Design B and C guides CAD rendering. Both guide designs were produced and used on the last 10 patients. (**C**) Plates CAD rendering. Teeth roots and inferior alveolar nerves were segmented and carefully avoided while planning the plates’ position. (**D**) Plate shown on a stereolithographic model of the planned mandibular anatomy. DMLS manufacturing allows for odd shaped plates. In this case the shape was designed to avoid interferences with the mental nerve. Single images joined in Adobe Photoshop.

The surgical plan was uploaded as a proprietary format (.ips for the planning, .spl for the splints) to the technical service through the IPS Gate web platform (*KLS Martin, Tuttlingen, Germany*). The original datasets (CBCT scan and digital models) were also uploaded. Subsequently, KLS Martin technical service designed the individualized mandible positioning guides, patient-specific plates and splints under the guidance of the surgeon. The positioning guides were designed to precisely guide the buccal and sagittal osteotomies, and the level of the lingual osteotomy, in order to reproduce the mutual relationship between the bony fragments according the points listed in the previous paragraph; screw trajectories were planned in order to avoid teeth roots and the inferior alveolar nerve (Fig. 2C). Three guide designs were used throughout the trial (Fig. 2A, B): Design A was built from selective laser sintered (SLS) polyamide, and was composed of a mandibular shell with a groove for the vertical osteotomy and a groove for the sagittal osteotomy; an inferior hook for the inferior border; holes for screw fixation of the guide (1.5 mm) and transfer of the custom-made plates (2.0 mm); an arm for teeth reference on the occlusal surface of the last two dental elements of the patient’s arch, in order to maximize accuracy and stability of the guide during surgery. Design B was analogous to design A except for the introduction of 7 mm long stainless-steel sleeves in transfer holes and the trimming of the most lingual part of the shell. Design C guides were made of direct metal laser sintered (DMLS) titanium alloy (Ti6Al4V), replacing the solid shell with a triangle mesh. The dental reference was replaced by a hook which came to an anterior stop against the last or second to last tooth of the patient’s dental arch; a detailed dental reference was avoided in this design due to possible guide inapplicability, production cost and time increase, and to limit the contact between titanium and teeth enamel. The custom titanium plates were designed to reposition the proximal and distal mandibular fragments in their planned positions, using the previously drilled holes (2.0 mm) for screw fixation as a reference. Plates were manufactured using titanium alloy (Ti6Al4V) Direct Metal Laser Sintering (DLMS) 3D printing (Fig. 2D); splints were manufactured using 3D printed dental resin. Digital three-dimensional models of bony fragments and plates were also provided by KLS Martin (in .STL format).

### Surgery

**Figure 3:**
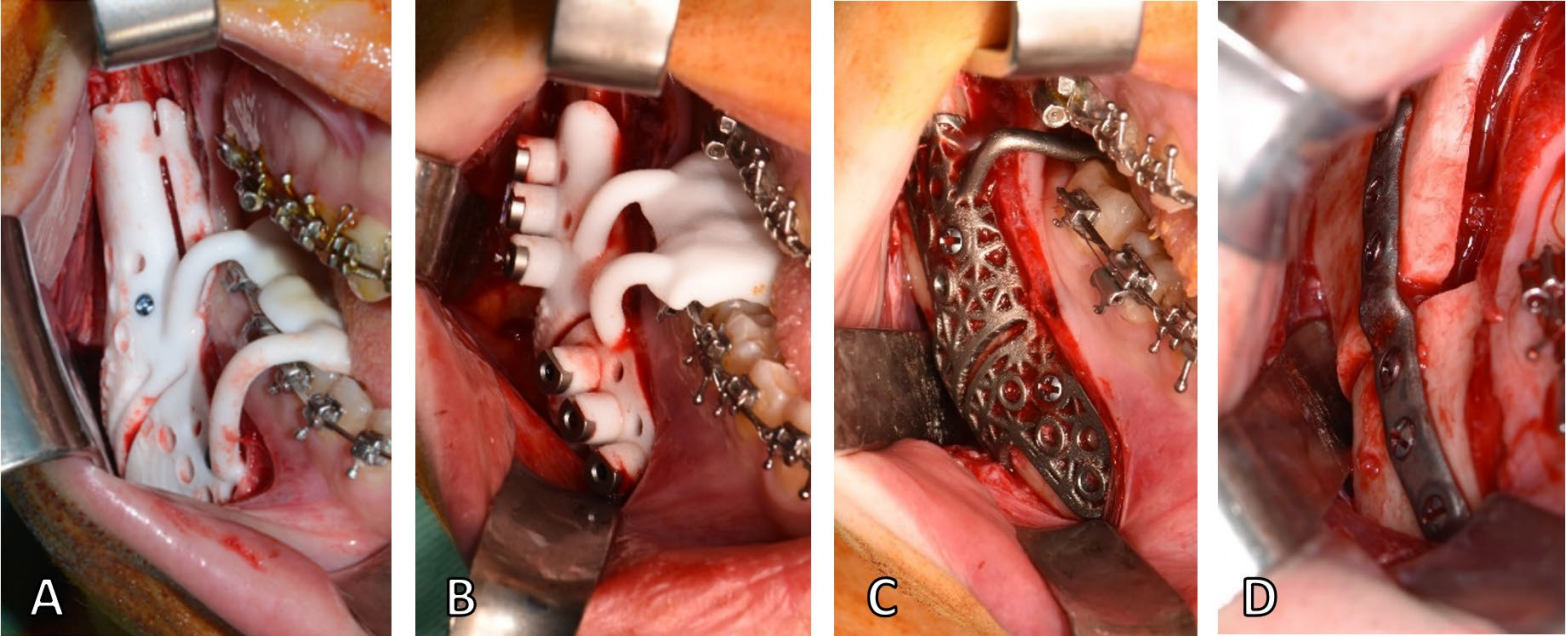
Intraoperative view of design A guide (**A**), design B guide (**B**), design C guide (**C**) and plate (**D**). Single images joined in Adobe Photoshop.

All patients were operated by the first author (GB) through a mandible-first approach, using the individualized system (Fig. 3). The bony surface of the mandible was exposed according to the conventional vestibular incision to perform BSSO. The guide was secured to the mandible with two titanium screws using the designed fixation holes (1.5 mm) to avoid any mobilization during the osteotomy. With the guide in place, the surgeon marked the osteotomy lines using ultrasonic bone-cutting tools (Piezo-Surgery, *Mectron SpA, Cerasco, Italy*) and drilled the transfer holes for the plate using an Angulus2 angulated drill (*KLS Martin, Tuttlingen, Germany*). Then the guide was removed to complete the osteotomy. Eventually, the proximal and distal fragments were fixed in the planned position using the patient-specific plates under the guide of pre-drilled transfer holes (2.0 mm). The intermediate CAD/CAM splint was used to maximize the stability of the teeth-bearing fragment while performing the osteosynthesis. The upper maxilla was managed according to the best vertical position and fixed using standard titanium manually bent miniplates and screws under the guide of the final CAD/CAM splint.

Design A guides were used on both sides of the mandible for the first 12 patients. For the last ten patients Design B and C guides were used following a split mouth procedure, using opposite guide designs on the same side for consecutive cases; once osteotomy lines were traced and screw holes were drilled under one guide type, the guide was removed and an analogous guide of opposite design was applied to cross-check holes and osteotomy lines alignment.

Intraoperative failures and complications were also assessed.

### Outcome evaluation

**Figure 4:**
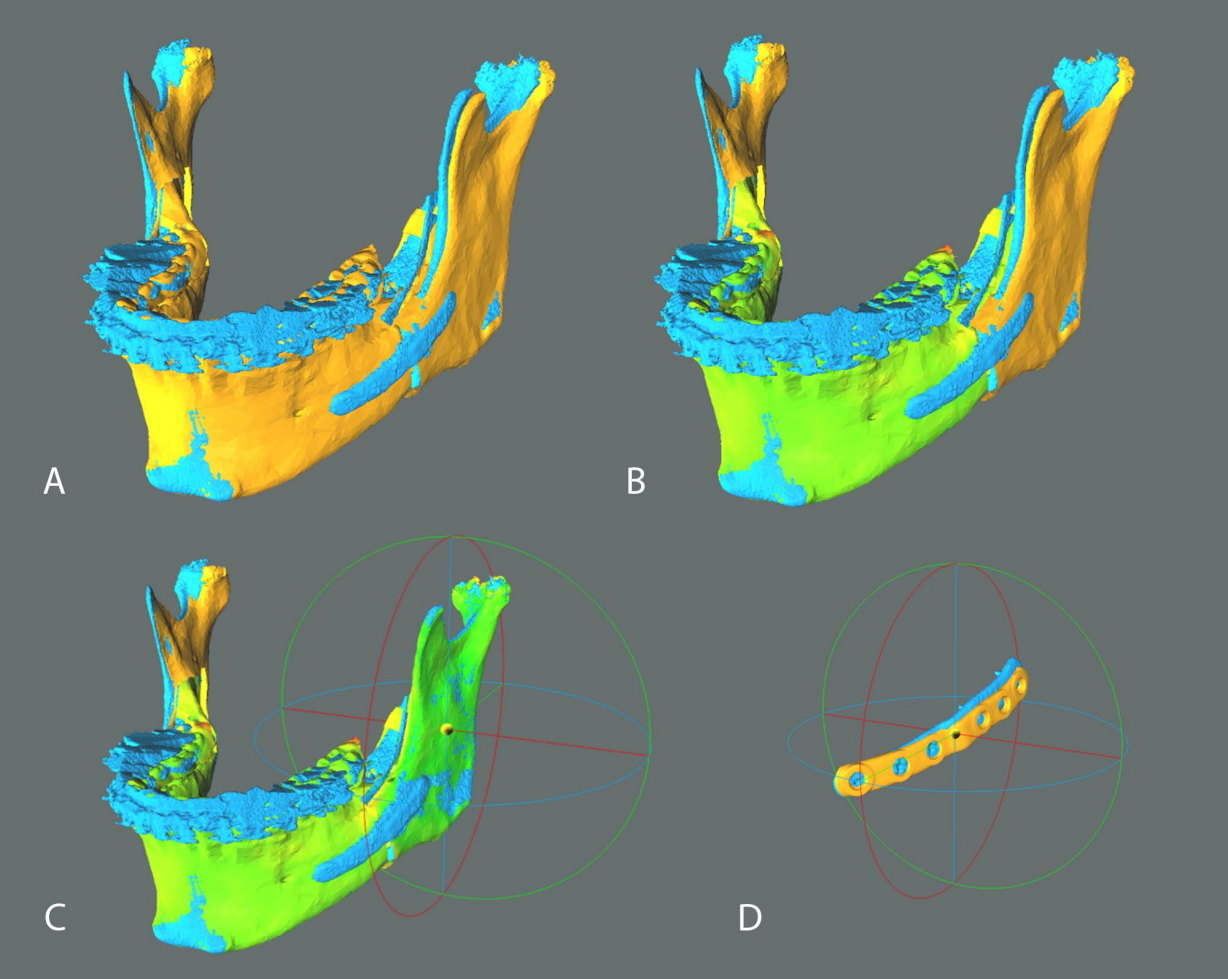
Analysis method in CloudCompare v2.9.1 software. Planned models (orange) were aligned to postoperative models (blue) on the basis of the teeth-bearing fragment (**A**). Colorimetric surface maps were used to check the alignment (**B**). Planned rami were aligned to postoperative rami position and the transformation was recorded; the alignment was visually checked via generation of colorimetric surface maps; axes and planes are also shown (X = red, Y = green, Z = blue) (**C**). An analogous protocol was applied to PSIs (**D**). Single images joined in Adobe Photoshop.

All patients underwent post-operative imaging with a CBCT-scan 1 month after surgery, before post-operative orthodontic treatment started and any tooth movements occurred (due to rigid orthodontics and daily use of the final splint), using the same machine and parameters of the pre-operative scan and maintaining the occlusion at maximum intercuspation. The post-operative DICOM dataset was processed to obtain a 3D model of the post-operative skull, mandible and mandibular plates with the software DICOM to Print (*3D Systems, Rock Hill, SC, USA*) and exported in STL format. In this early trial we focused on the mandible. Planned and post-operative meshes were compared using the open source CloudCompare software (*CloudCompare Project, cloudcompare.org*): the teeth-bearing fragments were registered with an iterative closest point (ICP) alignment method (Fig. 4A), and subsequently visually inspected via generation of colorimetric surface maps to check the alignment (Fig. 4B). We then evaluated the discrepancy between planned and obtained post-operative position of the mandibular plates and rami by analyzing the discrepancies in terms of rotation (roll, pitch and yaw) and translation (antero-posterior, lateral and vertical). These movements were determined by incorporating the segments in homologous bounding-boxes, aligning the planned model to the post-op result via ICP alignment and colorimetric map inspection (Fig. 4C, D), then evaluating the translational shift of the geometrical center and the rotational shift of the model according to Euler angles convention. The signed discrepancies were considered and tabulated according to the patient’s side (right or left) applying a mediolateral convention. Positive signed values identify forward, upward and lateral translations; a positive pitch angle identifies a clockwise rotation as seen from the patient’s right lateral aspect; a positive roll angle identifies a lateral displacement of the caudal margin of the ramus; a positive yaw angle indicates a lateral displacement of the anterior margin of the ramus. This convention allowed for comparison between opposite mandibular sides.

To obtain a comprehensive description of the angular and translational displacement of each 3D model considered, two further parameters were defined: total angular error and total translational error. The first is the angle in the axis-angle representation of a rigid body transformation, while the second is the translation vector module. Both measures are always positive by definition and were used to avoid positive and negative displacements canceling each other on average in the description of displacement.

The same protocol was applied to compare the preoperative CBCT-scan and the planned model, in order to quantify the planned three-dimensional shift for each mandibular ramus in respect to the pre-operative position. To accomplish this evaluation, the ICP alignment was carried out referring to the cranial base as fixed reference.

Post-operative failures and complications were also assessed.

### Statistical analyses

Given the limited sample size (n = 44), all measures were summarized using median and interquartile range (IQR). However, to allow comparison with the existing literature, mean and standard deviation were also provided for each measure. Boxplots were used to display the frequency distribution of the measures. Non-parametric two-tailed tests were used in all cases due to limited sample size and theoretical asymmetric distribution of total angular and translational errors.

Because in a preliminary analysis (Mann–Whitney U-Test) performed on the first 12 patients no significant difference was found in the precision of rami and plate positioning between the left and right sides, rami and plates were considered as separate entities, without reference to the side.

Spearman’s correlation coefficient (n = 44) was used to examine the extent to which inaccuracies in the final position of the plate were associated with inaccuracies in the position of the ramus. Similarly, the correlation between the pre-operative to planned rami transformations and the inaccuracy in the final position of the rami was investigated, in order to determine whether greater planned displacements could increase the inaccuracy of the outcome.

Kruskal–Wallis test (n = 44) followed by post-hoc pairwise comparisons was carried out to analyse differences in the post-operative position of rami and plates among guide designs.

IBM SPSS Statistics 25 (*IBM Corp., Armonk, NY, USA*) was used to perform the analyses. For all tests the significance level was set to α = 0.05.

## Results

**Figure 5:**
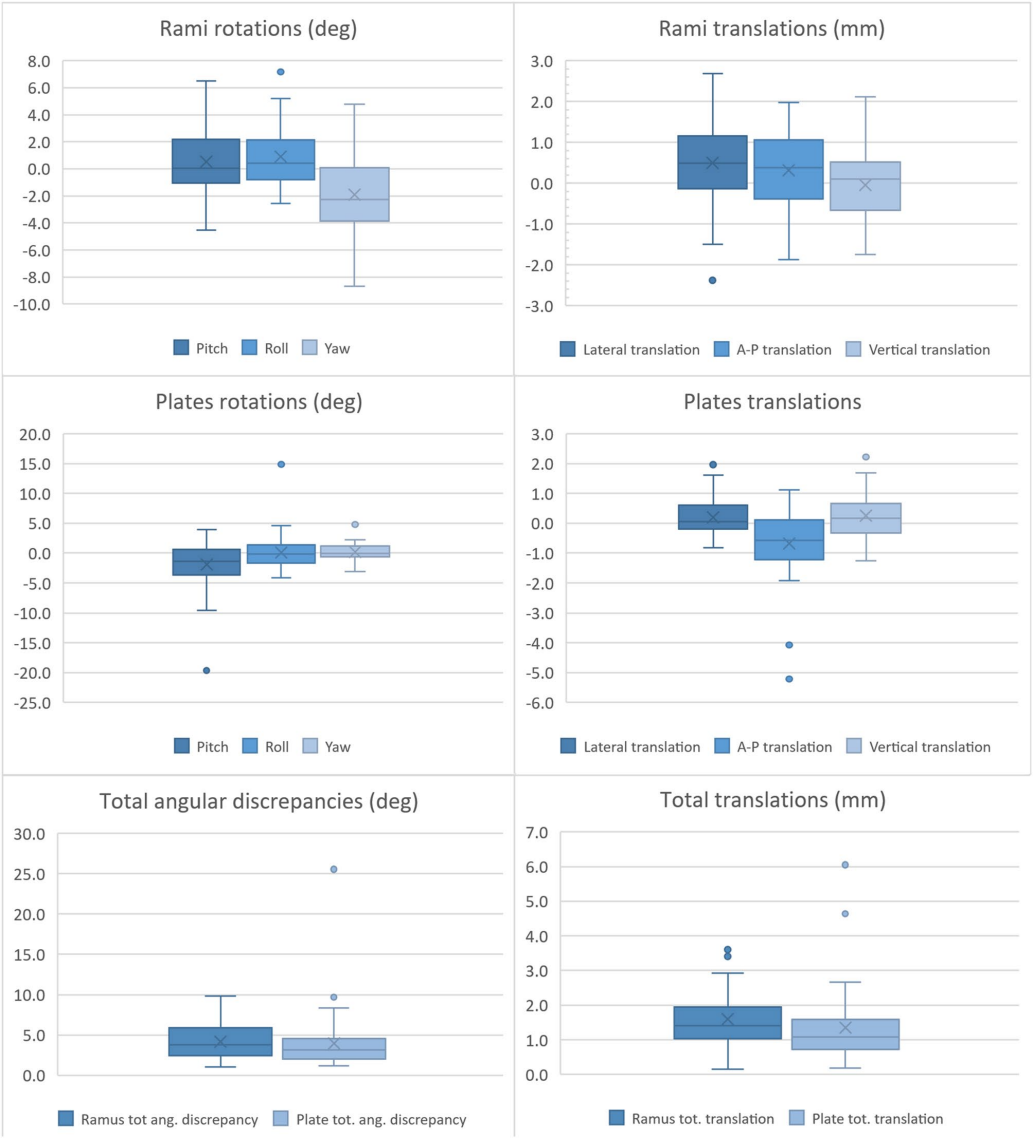
Box plots displaying the eight considered variables for both rami and plates (n = 44).

Intraoperatively, the system was successful in all cases, without the need to shift to the conventional method with the manually bent titanium stock plates. From a clinical point of view, the result in terms of verticality, incisal midline alignment, incisal exposure and overall symmetry was deemed satisfactory in all patients. The average follow-up is 14 months (range 5–29). The frequency of postoperative adverse events is reported in Table 1. Descriptive statistics are reported in Table 2. Discrepancies between the planned and obtained post-operative position of the plates and rami are plotted in Fig. 5. Rotational and translational displacement values of all cases are reported in Supplementary Dataset 1.

**Table 1:**
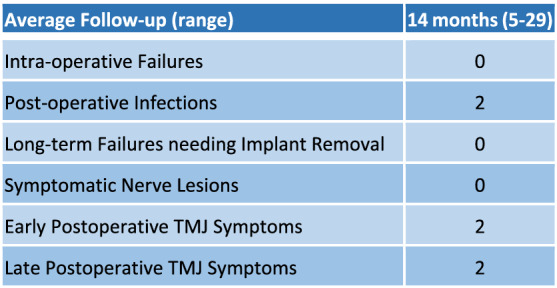
Frequency of postoperative adverse events.

**Table 2:**
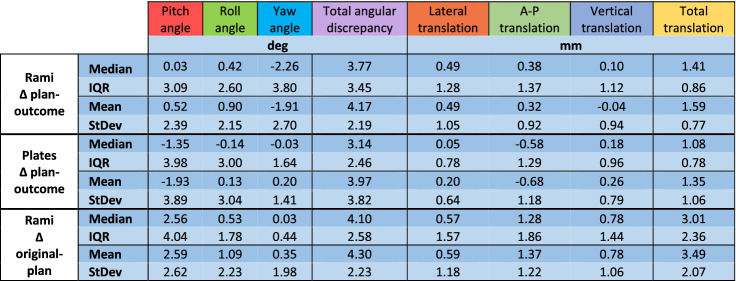
Median, interquartile range, mean and standard deviation of each variable considered.

**Figure 6:**
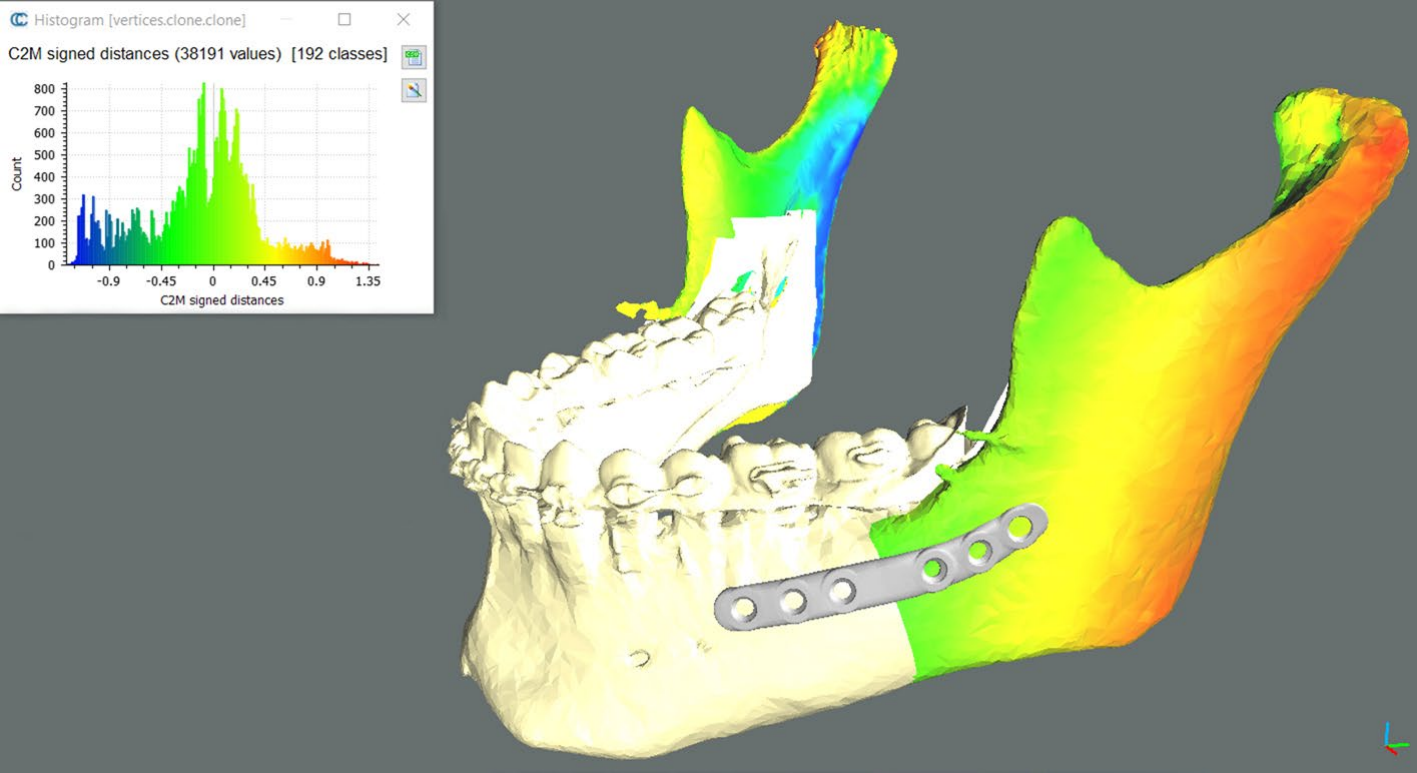
Simulation of the obtained median rami discrepancies on an example surgical plan in CloudCompare v2.9.1 software. A colorimetric surface map was used to show surface differences of the simulated median position from planning. Negative yaw can be seen as the prominent component of the median discrepancy.

In regard to the rami positioning, we obtained a median total angular error of 3.77° (IQR 3.45°) and a median total translational error of 1.41 mm (IQR 0.86 mm). Except for the yaw angle (median − 2.26°, IQR 3.81°) median signed rotational discrepancies were within 0.5° from the planned position and respective IQRs were below 4°; median signed translational discrepancies remained within 0.5 mm from the planned position (mediolateral translation being the furthest from planned at + 0.49 mm) with IQRs below 1.5 mm. Median rotational and translational discrepancies were applied to an example case planning for graphical representation. A colorimetric surface map mapping distances between the simulated median result and surgical plan was calculated and applied to the displaced rami (Fig. 6).

In plates positioning, the median total angular error was 3.14° (IQR 2.46°) and the median total translational error was 1.08 mm (IQR 0.78 mm). Apart from pitch (median − 1.34°, IQR 3.98°), median signed rotational discrepancies were within 0.15° from planned position with IQRs below 4°. Signed translational discrepancies were within 0.6 mm from planned position (anteroposterior being the furthest at − 0.58 mm) with IQRs below 1.5 mm.

Spearman’s correlation coefficients are reported in Tables 3a and b.

**Table 3:**
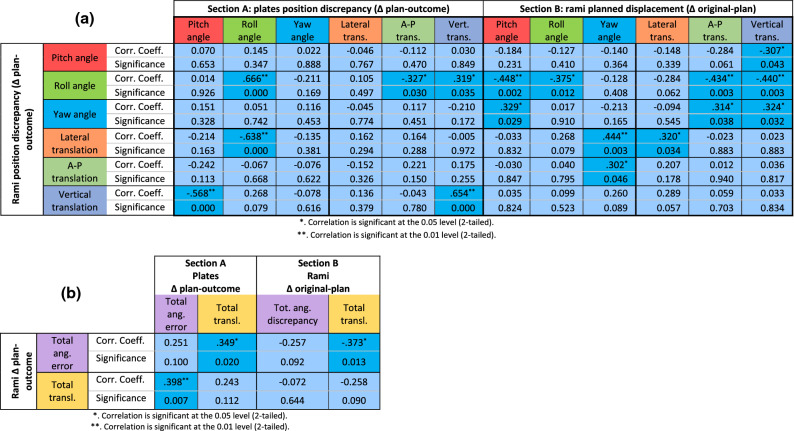
Spearman’s correlation coefficients of study variables (n = 44). Significant correlations are highlighted in turquoise.

In (a), Section A shows the correlations between the signed discrepancies of the planned and obtained positions of the rami and the signed discrepancies of the planned and obtained positions of the plates; Section B shows the correlations between the signed discrepancies of the planned and obtained positions of the rami and the signed discrepancies of the planned and preoperative positions of the rami.

In (b), Section A shows the correlations between rami and plates total discrepancies between obtained and planned position; Section B shows the correlations between rami total obtained-from-planned discrepancies and original-to-planned discrepancies.

Strong significant positive correlations were found between plate and ramus roll angle (0.666, p < 0.001) and between plate and ramus vertical translation (0.654; p < 0.001). Strong negative correlations were found between rami vertical translation and plates pitch angle (− 0.568; p < 0.001), and between rami mediolateral translation and plates roll angle (− 0.638; p < 0.001). Weaker significant correlations were found between rami roll angle and plate both anteroposterior (− 0.327; p = 0.03) and vertical (0.319; p = 0.035) translation. In terms of precision, positive correlations were found between rami total angular error and plates total translational error (0.349; p = 0.02) and between rami total translational error and plates total angular error (0.398; p = 0.007).

Both positive and negative significant correlations were found between rami original-to-plan displacement and rami plan-to-outcome displacement, across all rotational and translational values. Since only rotational transformations pivoted on the condylar head were used to change the position of the rami during planning, original-to-plan translations were treated as a byproduct of the change of pivotal point in our analysis method. Negative correlations were found between plan-to-outcome rami roll angle and planned roll and pitch angles (− 0.448; p = 0.002 and − 0.375; p = 0.012 respectively); planned pitch angle was also positively correlated to plan-to-outcome yaw angle (0.329; p = 0.029). Planned yaw angle was positively correlated to planned-to-outcome lateromedial and anteroposterior rami translation (0.444; p = 0.003 and 0.302; p = 0.046 respectively). Bounding box center translation of the rami during planning negatively correlated with rami total angular outcome error (− 0.373; p = 0.013). The total original-to-plan angle correlation with total plan-to-outcome angular error was similarly negative (− 0.257; p = 0.092), although it failed to reach statistical significance.

No significant difference in both positional tendency and precision of the ramus positioning was found among guide designs (Table 4) , while significant differences were found in final position of the plate (mediolateral translation p = 0.015, anteroposterior translation p = 0.005) , with Designs A, B and C leading to a respective median mediolateral translation of + 0.51 mm, − 0.06 mm and − 0.26 mm and a respective median anteroposterior translation of − 1.03 mm, − 0.19 mm and + 0.07 mm.

**Table 4:**
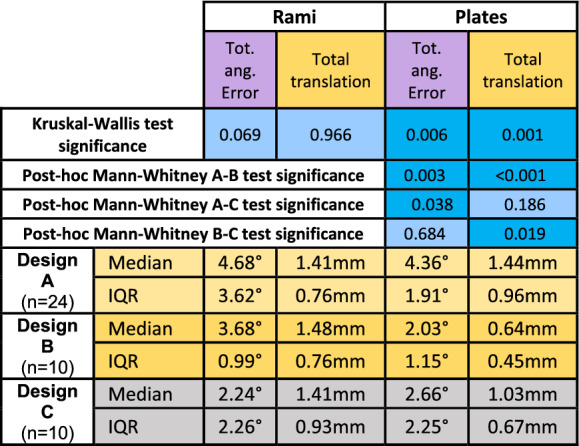
p-values of the Kruskal–Wallis test (n = 44) with pairwise post-hoc Mann–Whitney tests comparing plates and rami total angular and translational discrepancy among guide designs.

Medians and IQRs for each variable obtained with different guide designs are also shown.

Significant differences were also found in plates positioning precision values (total angular error p = 0.006, total translational error p = 0.001). Post-hoc significant differences (p < 0.01) were found between Designs A and B in total angular error and total translational error. Designs A to C differed in total angular error (p = 0.038), and Designs B to C in total translational error (p = 0.019).

Design B showed the lowest median rotational error, (2.03 mm vs. Design A = 4.35 mm and Design C = 2.65 mm) and translational error (0.64 mm vs. Design A = 1.44 mm and Design C = 1.02 mm).

## Discussion

In regard to the use of PSIs in mandibular orthognathic surgery, the largest cohort treated with mandibular PSIs is the one described by Suojanen et al., (n = 30)^9^, in which the only outcome considered was the clinical applicability of PSIs, and no evaluation of surgical plan transfer accuracy has been performed. The most extensive precision analysis on patients treated with PSIs was carried out by Li et al.^8^, who determined the planned to post-operative precision of the mandibular rami positioning with a method similar to our own (n = 10).

While Brunso et al.^10^ reported the use of different guide designs, no outcome precision comparison was carried out between cases treated with different designs and, to our knowledge, no study reported this kind of comparison and/or assessed plate positioning.

A bimaxillary PSI-guided splintless procedure, as reported by Li et al.^8^, allows minimal to no planning error and intraoperative plan correction, resulting in possible PSI inapplicability. Furthermore, the rigidity of sintered titanium alloy makes the modification of custom-made implants almost impossible.

A mandible-first mandibular PSI-guided splinted procedure, while requiring accurate planning of the optimal mandibular anatomy and prediction of bony segment interferences along the osteosynthesis surface, remained flexible enough to allow for intraoperative verticality correction according to aesthetic principles. The overall accuracy in full face virtual plan transfer to the patient will be analysed in a separate study. A further study will investigate the post-operative condylar position and morphology, once all patients will have reached a minimum follow-up of 9 months.

As to the mandibular anatomical outcome, discrepancy from planning was small for all considered variables. The central tendency to a negative yaw discrepancy found can be attributed to not completely manageable BSSO osteosynthesis interface interferences.

Plates positioning was also accurate, and the central tendency to a positive pitch angle can be attributed to a tendency of the guides to assume a similarly pitched position due to a lack of features of the mandible’s buccal aspect.

Notably, not all plate discrepancies from the designed position were directly correlated to a similar discrepancy in the ramus position, with roll angle and vertical translation being the only ones positively correlated between rami and plates. Plates rotation to rami translation negative correlations can be geometrically explained with a shift in the position of the ramus’ bounding box center, and a similar interpretation can be given to the correlations between rami roll angle and plate anteroposterior and vertical translations.

Plates and rami precision values (total angular error and total translational error) were cross correlated but not directly correlated, although both direct correlation coefficients reached values close to 0.25, with p-values near 0.1.

In respect to the correlation between rami displacement during planning and outcome error, both positive and negative correlations were found. Firstly, a negative correlation was found between rami total angular error and total translation of the planned ramus, which can be interpreted as an increase in error in case of excessively conservative ramus planned position, which in turn can lead to unplanned and less manageable bony interference. A similar trend can be observed between total angular displacement of the ramus from the original position and total angular error in post-operative ramus position, although this correlation does not reach statistical significance. The roll angle appears to be the most sensitive to excessively conservative ramus correction, while ramus yaw angle correction is positively correlated to a lateral and anterior translation, possibly due to imperfect simulation of the patient’s TMJ mechanical behavior in the planning software. Similarly, a positive correlation was found between roll correction and lateral translation, though not reaching statistical significance (p = 0.079).

The weak correlation between plate and ramus position can partially explain why, while one guide design (B) led to a significantly more precise plate positioning, ramus positioning was not affected by different guide designs. In our interpretation, one factor that may have contributed to the increased plate positional accuracy with Design B guides are the transfer hole sleeves: while increasing the bulk of the guide, the sleeves’ lower diameter tolerance helped center the drill in the transfer holes. From a surgical point of view, Design C guides were the easiest to position and, due to the mesh design, it was easier to check if the guide was fully in contact with the mandible. The reduced bulk made drilling holes and tracing osteotomy lines easier.

These findings suggest that PSI system inaccuracies may not be the main determinant in proximal segment positioning error, with bony segments interference, segmentation, software and planning inaccuracies, implants and tools production tolerances all playing a role. The largest positional error in the positioning of a plate (total angular error = 25.5°, total translational error = 6 mm) was obtained in a patient in which the corresponding ramus did not notably shift from the planned position. This finding could be explained by a certain degree of error compensation that may be intrinsic to the PSI system or may be due to the operator’s surgical experience and virtual planning knowledge.

## Conclusion

Our findings suggest that the mandible-first mandibular PSI-guided procedure is accurate when transferring the virtually planned mandibular anatomy to the patient. Different positioning guide designs did not affect the precision of mandibular anatomy reproduction, however Design B guides led to a slightly more precise plate positioning, which in turn could contribute to an overall more accurate procedure. Given the limited sample size, further precision differences between the guides cannot be ruled out; until a larger cohort of patients is enrolled, the choice between the different guide types analyzed can be determined solely by cost and surgeon preference.

Among the many factors that contribute to the outcome in a PSI-guided procedure, one that needs to be accurately planned and managed is the interference between teeth-bearing fragment and condyle-bearing fragment. Our data show that an excessively conservative ramus position planning might lead to increased interference and outcome inaccuracy. Imperfect software simulation of TMJ soft tissue during planning can add to outcome inaccuracy. Anyway, while all the aforementioned factors need to be taken into account in PSI case planning and surgery, the limited inaccuracies of the system appear to be well compensated and the outcome was clinically satisfactory in all patients.

## Supplementary information

Supplementary Dataset

Supplementary Information
