# Supplementary material for: Validation of a patient-specific system for mandible-first bimaxillary surgery: ramus and implant positioning precision assessment and guide design comparison
Source: Sci Rep. 2020 Aug 7;10:13317. doi: 10.1038/s41598-020-70107-w (PMC7415134; doi:10.1038/s41598-020-70107-w)
Supplement: Supplementary file 2 — Supplementary Information [file 41598_2020_70107_MOESM2_ESM.docx]

**Supplementary Material**

“Supplementary Dataset.xslx”

**Title**: “Complete table of rotational and translational analysis results”

**Authors**:

Giovanni Badiali,

Mirko Bevini,

Elisa Lovero,

Elisabetta De Simone,

Paola Rucci.
